# Complete mitochondrial genome of the mud loach *Misgurnus mizolepis* (Cypriniformes, Cobitidae) and its phylogenetic position in the Cypriniformes

**DOI:** 10.1080/23802359.2016.1247675

**Published:** 2016-11-11

**Authors:** Seungki Lee

**Affiliations:** Biological and Genetic Resources Assessment Division, National Institute of Biological Resources, Incheon, Republic of Korea

**Keywords:** Cypriniformes, Cobitidae, *Misgurnus mizolepis*, mud loach

## Abstract

The mud loach (*Misgurnus mizolepis*) is a small benthic species belonging to the family Cobitidae. In this study, I report the first sequencing and assembly of the complete mitochondrial genome of *M. mizolepis*. The complete mitochondrial genome is 16,647 bp long, consisting of 13 protein-coding genes, 22 tRNA genes, 2 rRNA genes, and a control region. It has the typical vertebrate mitochondrial gene arrangement. Phylogenetic analysis using mitochondrial genomes of 20 species showed that *M. mizolepis* is clustered with *M. anguillicaudatus* and *M. bipartitus*. This mitochondrial genome provides potentially important resources for studying molecular evolution and biogeography.

The mud loach, *Misgurnus mizolepis* (Günther 1888), is a small benthic species belonging to the family Cobitidae. This species is widely distributed in midstream and downstream regions in Korea, China, and Taiwan (Kim & Park 2007). It is an ecologically and commercially important species and is extensively used as a model animal for biological research (Kim et al. 1994; Nam et al. 2001). To the best of our knowledge, this is the first study to determine the complete mitochondrial genome of *M. mizolepis*, and to analyze the phylogenetic relationship of this species among Cypriniformes fishes.

The *M. mizolepis* specimen (standard length, 87.7 mm) was collected from the Yeongam-gun, South Korea (34.49N, 126.39E). The specimen was deposited in the National Institute of Biological Resources (NIBR, Voucher No. NIBRGR0000098717). Genomic DNA from muscle tissue was sequenced and assembled using the Illumina Hiseq 4000 sequencing platform (Illumina, San Diego, CA) and *SOAPdenovo* assembler at Macrogen Inc. (Korea), respectively. The complete mitochondrial genome was annotated using MacClade ver. 4.08 (Maddison & Maddison 2005) and DNASIS ver. 3.2 (Hitachi Software Engineering). Experiments were conducted in accordance with the Guidelines of Animal Ethics published by the NIBR.

The complete mitochondrial genome of *M. mizolepis* (GenBank accession no. AP017654) is 16,647 bp long and includes 13 protein-coding, 22 tRNA, and 2 rRNA genes. The *ND6* gene and eight tRNA genes are encoded on the light strand. The overall base composition of the heavy strand is 29.74% A, 25.50% C, 16.38% G, and 28.37% T. As in mitogenomes of other vertebrates (Saccone et al. 1999), the AT content is higher than the GC content. All tRNA genes can fold into a typical cloverleaf structure and are 66–76 bp long. The 12S rRNA (953 bp) and 16S rRNA genes (1679 bp) are located between tRNA^Phe^ and tRNA^Val^ and between rRNA^Val^ and tRNA^Leu(UUR)^, respectively. Of the 13 protein-coding genes, 12 start with ATG; the exception being *COI*, which starts with GTG. Six of the 13 protein-coding genes terminate with incomplete stop codons, T (*ND2*, *COIII*, *ND3*, and *Cytb*) and TA (*ATP6* and *ND4*), whereas the remaining seven genes ended with complete stop codon (TAA). A control region (922 bp) is located between tRNA^Pro^ and tRNA^Phe^.

Phylogenetic trees were constructed (maximum likelihood) with 1000 replicates using MEGA 7.0 software (MEGA, PA, USA) (Kumar et al. 2016) for the newly sequenced mitogenome and a further 19 complete mitogenome sequences downloaded from the National Center for Biotechnology Information. We confirmed that *M. mizolepis* is clustered with *M. anguillicaudatus* (Miya et al. 2015) and *M. bipartitus* (Huang et al. 2015) and grouped with the other Cobitidae species (Figure 1). This mitochondrial genome provides potentially important resources for studying molecular evolution and biogeography.

**Figure 1.**
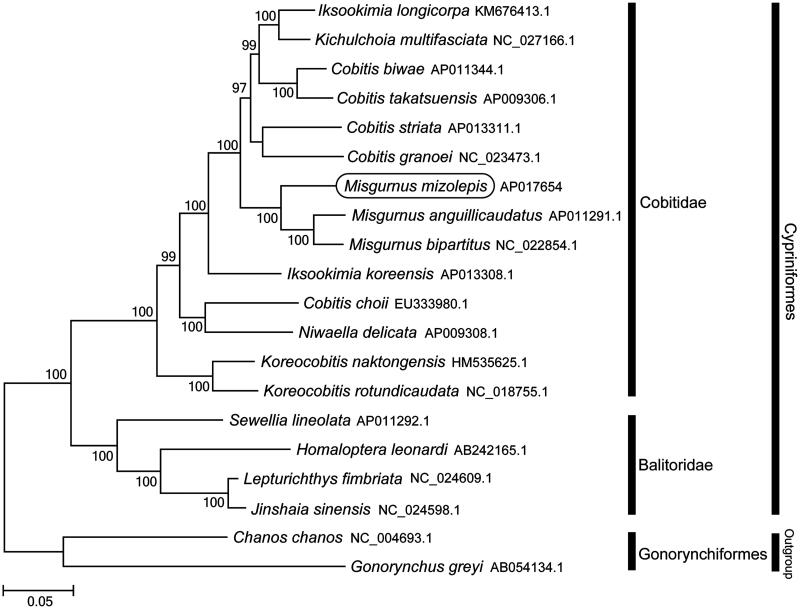
Phylogenetic position of *Misgurnus mizolepis* based on a comparison with the complete mitochondrial genome sequences of 17 Cyprinidae species and 2 outgroup species. The analysis was performed using the MEGA 7.0 software. The accession numbers for each species are indicated after the scientific names.
